# Mesenchymal Stem Cell-Derived Exosomes: Immunomodulatory Evaluation in an Antigen-Induced Synovitis Porcine Model

**DOI:** 10.3389/fvets.2017.00039

**Published:** 2017-03-21

**Authors:** Javier G. Casado, Rebeca Blázquez, Francisco Javier Vela, Verónica Álvarez, Raquel Tarazona, Francisco Miguel Sánchez-Margallo

**Affiliations:** ^1^Stem Cell Therapy Unit, “Jesús Usón” Minimally Invasive Surgery Centre, Cáceres, Spain; ^2^Immunology Unit, Department of Physiology, University of Extremadura, Cáceres, Spain; ^3^CIBER de Enfermedades Cardiovasculares, Cáceres, Spain

**Keywords:** exosomes, mesenchymal stem cells, synovitis, immunomodulation, inflammation

## Abstract

Synovitis is an inflammatory process associated with pain, disability, and discomfort, which is usually treated with anti-inflammatory drugs or biological agents. Mesenchymal stem cells (MSCs) have been also successfully used in the treatment of inflammatory-related diseases such as synovitis or arthritis. In the last years, the exosomes derived from MSCs have become a promising tool for the treatment of inflammatory-related diseases and their therapeutic effect is thought to be mediated (at least in part) by their immunomodulatory potential. In this work, we aimed to evaluate the anti-inflammatory effect of these exosomes in an antigen-induced synovitis animal model. To our knowledge, this is the first report where exosomes derived from MSCs have been evaluated in an animal model of synovitis. Our results demonstrated a decrease of synovial lymphocytes together with a downregulation of TNF-α transcripts in those exosome-treated joints. These results support the immunomodulatory effect of these exosomes and point out that they may represent a promising therapeutic option for the treatment of synovitis.

## Introduction

Osteoarticular disorders are the major cause of disability in western countries causing pain, discomfort, disability, and affecting the quality of life of millions of people. Osteoarticular disorders are usually linked to joint inflammation and accompanied by redness, swelling, and pain. This local inflammation is provoked by different causes such as trauma, injuries, microorganism infections, or autoimmune disorders such as rheumatoid arthritis. The inflammation of the synovium around a joint, also called synovitis, is frequently observed in the early phase of osteoarthritis (1) and in clinically active rheumatoid arthritis patients (2). One of the consequences of persistent synovitis is the cartilage matrix degradation (3, 4) and alterations in chondrocyte function. Moreover, synovitis causes hypoxia and acidity in synovial fluid (SF) and subchondral bone (5) and enhances angiogenesis (6).

The treatments to reduce pain and swelling for transient synovitis includes anti-inflammatory agents such as non-steroidal anti-inflammatory drugs (NSAIDs) and/or corticosteroids (7). Non-pharmacological treatments such as hip aspiration (8) as well as rest, ice, compression, and elevation are extremely helpful and effective in the treatment of synovitis (9). Additionally, intrasynovial injections of biologically based therapies such as platelet-rich plasma (10) and autologous conditioned serum (11) have been found to be very effective.

Local or systemic administration of mesenchymal stem cell (MSC)-based therapies has recently emerged as a promising therapeutic approach for the treatment of inflammatory-related diseases (12). These cells have an immunomodulatory potential on cells of both the innate and adaptive immune system and recent clinical trials have demonstrated very promising results for the treatment of osteoarticular diseases (13, 14). In the case of synovitis, the therapeutic use of MSCs has also been evaluated in veterinary medicine, specifically in horses with intra-articular injections of xenogeneic, allogeneic, and autologous MSCs (15). Moreover, in a recent randomized and blinded study using a LPS-induced synovitis model, equine allogeneic umbilical cord blood-derived mesenchymal stromal cells could reduce the nucleated cell counts in SF (16).

Accumulative evidences have established that the effect of MSC transplantation is thought to be mediated, in part, by a paracrine effect. In this sense, the use of exosomes derived from MSCs (hereinafter referred exo-MSCs) has become a promising tool for the treatment of inflammatory-related diseases (17–19).

Exosomes are small membranous vesicles secreted by most cell types. These vesicles participate in cell–cell communication and their content consists on RNA, lipids, and proteins. Some of these proteins (i.e., CD9, CD63, or CD81) are ubiquitously expressed, but depending on the cell source, cell type-specific proteins can be found being responsible of their functionality (20). The proteins, lipids, and RNA expression of exosomes from different cells and organisms are extensively described in ExoCarta database (21). Previous studies from our group have reported that exo-MSCs exerted an immunomodulatory potential against *in vitro* activated T cells (22). Additionally, several evidences have shown that exo-MSCs could play active roles in promoting angiogenesis (23), antiapoptotic effect (24, 25) as well as in cell proliferation (26).

In the last years, the therapeutic potential of exo-MSCs has been demonstrated in disease-specific animal models. Very promising results have been obtained in small animal models for the treatment of cardiovascular diseases where exo-MSCs showed a reduction of myocardial ischemia/reperfusion injury (27). In renal fibrosis, where the microRNA-let7c secreted by the exosomes attenuated renal fibrosis (28). In wound healing, where released exosomes promoted angiogenesis (29). In necrotizing enterocolitis, where exosomes from bone marrow-derived stem cells protected the intestines (30). In acute lung injury, where the exosomes maintain the functional phenotype of the parent cell (31). In postischemic neurological impairment, where extracellular vesicles induce long-term neuroprotection, neuroregeneration, and neurological recovery (32). Finally, it is important to note that, although the therapeutic effect of exo-MSCs has been widely studied in small animals, only a few studies have evaluated their therapeutic effect in large animal models (33, 34).

In summary, although the therapeutic effect of MSCs in osteoarticular diseases is widely accepted, the hypothetical beneficial effect of exo-MSCs in joint inflammation has not been evaluated. This paper aimed to evaluate the immunomodulatory effect of exo-MSCs in a clinically relevant animal model of antigen-induced synovitis. The analysis of leukocytes, lymphocytes, and inflammatory cytokines in SF revealed a potential therapeutic effect of exo-MSCs in the setting of inflammatory and osteoarticular disorders.

## Materials and Methods

### Animals and Ethical Issues

Eight large white pigs were housed in the animal facility at the Minimally Invasive Surgery Center and used for all experimental procedures. Animals aged 3 months and weighed 25–35 kg at the beginning of the study were used. All experimental protocols were approved by the Committee on the Ethics of Animal Experiments of Minimally Invasive Surgery Center and fully complied with recommendations outlined by the local government (Junta de Extremadura) and by the Directive 2010/63/EU of the European Parliament on the protection of animals used for scientific purposes.

### Immunization Protocol and Antigen-Induced Synovitis

For animal immunizations, a solution with 20 mg/ml of BSA (Sigma-Aldrich, St. Louis, MO, USA) was prepared and passed through a 0.2-μm sterilized microfilter. An equal volume of Freund Complete Adjuvant (Sigma-Aldrich, St. Louis, MO, USA) was mixed with the BSA solution and emulsified. The immunization was performed by subcutaneous injections of this emulsion. A total of 0.4 ml/kg was injected on days 0, 14, and 21. On day 28, a total of 0.5 ml of SF was aspirated from carpal joints. Intra-articular injections of BSA (0.5 ml at 20 mg/ml) were bilaterally performed to induce an antigen-mediated immune response. The left carpal joints were used as control (BSA co-administered with PBS) and the right carpal joints were used for exosome-based treatments (BSA co-administered with exosomes). The exosomes were used at the concentration of 500 μg protein/injection in a total volume of 500 μl.

### Anesthetics Procedures

Every procedure was performed under anesthesia. For blood sampling and subcutaneous BSA injections, anesthesia was induced by intramuscular injection of 10 mg/kg ketamine hydrochloride and 0.02 mg/kg dexmedetomidine hydrochloride. The animals were recovered with 0.02 mg/kg atipamezole hydrochloride. For SF sampling, anesthesia was induced by the same procedure together with an intravenous bolus injection of 2 mg/kg propofol and 3 mg/kg of tramadol. According to ethical and animal welfare concerns, all the animals received analgesic treatment with a solution of buprenorphine hydrochloride at 0.3 mg/ml and 0.03 ml/kg for 7 days after intra-articular injection.

### Quantification of Anti-BSA Antibodies by ELISA

In order to quantify the anti-BSA IgG titers on immunized animals, an ELISA test was performed on plasma samples at days 0, 7, 14, 21, and 28. Microplate coating was performed by an overnight incubation with BSA at 20 μg/ml. The coating solution was washed twice with 200 μl of PBS/Tween-20 (0.05%, 7.4 pH). In order to prevent the non-specific binding of the antibodies, the remaining protein-binding sites were blocked by adding 200 μl of BSA and incubated at 4°C for 2 h. The microplate was washed four times with 200 μl PBS/Tween-20. Plasma samples were diluted 1:200 with PBS and 100 μl of this dilution was added to each well. The plate was incubated at 4°C for 2 h. After washing four times with PBS/Tween-20, 100 μl of 1/5,000 diluted horseradish peroxidase-conjugated secondary antibody (Rabbit Anti-Pig IgG, Thermo Fisher Scientific, Waltham, MA, USA) was added to each well and the plate was incubated at 4°C for 2 h. Finally, the plate was washed four times and 100 μl of 3,3′,5,5′-Tetramethylbenzidine (Sigma-Aldrich) was added to each well. Then, 2 min later, 100 μl of 1M HCl was added to stop the reaction. Absorbance was measured at 450 nm on a Synergy Mx spectrophotometer (BioTech Industries, Newton, NC, USA) and quantified related to the baseline.

### Isolation and Expansion of Porcine Bone Marrow-Derived MSCs

Bone marrow-derived MSCs were harvested from the iliac crest from anesthetized large white pigs. The mononuclear cells were isolated by centrifugation over Histopaque-1077 (Sigma, St. Louis, MO, USA). Mononuclear cells were recovered and washed twice with PBS. Finally, mononuclear cells were re-suspended in DMEM containing 10% FBS (Sigma), 5 μl/ml fungizone, and 1% Penicillin/streptomycin (Lonza BioWhittaker™, Basel, Switzerland). Cells were seeded onto tissue culture flasks and expanded at 37°C and 5% CO_2_. Following 48 h of culture, the non-adherent cells were removed. Adhered cells were passaged at 80–90% confluence by trypsinization (0.25% trypsin solution) and seeded to a new culture at a density of 5,000 cells/cm^2^. Culture medium was changed every 3–4 days.

The porcine MSCs were phenotypically and functionally characterized by flow cytometry and *in vitro* differentiation assays. Cells were positive for CD29, CD44, CD90, CD105, and SLA-I and negative for CD45 and SLA-II. They also showed their ability to differentiate toward adipogenic, osteogenic, and chondrogenic lineages (35).

### Isolation, Purification, and Characterization of MSC-Derived Exosomes

The MSCs-derived exosomes (exo-MSCs) were obtained from porcine bone marrow-derived MSCs cultured in 175 cm^2^ flasks. When cells reached a confluence of 80%, culture medium (DMEM containing 10% FBS) was replaced by exosome isolation medium (DMEM containing 1% insulin-transferrin-selenium). The supernatants were collected every 3–4 days. To eliminate dead cells and debris, the supernatants were centrifuged at 1,000 × *g* for 10 min and 5,000 × *g* for 20 min at 4°C. About 15 ml of these supernatants were ultra-filtered through 3 kDa MWCO Amicon^®^ Ultra devices (Merck-Millipore, MA, USA). Samples were spun at 4,000 × *g* for 60 min and 200–300 μl of concentrated supernatant was collected and stored at −20°C. Prior to *in vivo* experiments, the proteins were quantified by Bradford assays, a very common method to indirectly quantify exosomes (36–39). The concentration and size of the particles were measured by nanoparticle tracking analysis (NanoSight Ltd., Amesbury, UK) that relates the rate of Brownian motion to particle size. Results were analyzed using the software package version 2.2. Triplicate samples were diluted 1:10 in sterile-filtered PBS and analyzed. The mean size of isolated vesicles ranged from 150 to 200 nm (Figure S1A in Supplementary Material).

For flow cytometric analysis by fluorescent activated cells sorting, exosomes were conjugated with latex beads as previously described (40). Briefly, 5 μg of exosomes were incubated with 10 μl of latex beads 15 min at room temperature. After, PBS was added to a final volume of 1 ml and samples were incubated overnight at 4°C. Finally, 110 μl of 1M glycine were added to each tube. After 30 min of incubation, samples were centrifuged, washed, and re-suspended in a final volume of 0.5 ml PBS/0.5% BSA. These exosomes-coated beads were incubated for 1 h at room temperature with appropriate concentrations of monoclonal antibodies (mAbs) in the presence of PBS containing 0.5% BSA. The exosomes-coated beads were stained with FITC-conjugated human mAbs against CD44 and CD90 (porcine cross-reactive) from Serotec (Kidlington, UK). After incubation with antibodies, the exosomes-coated beads were washed and re-suspended in PBS/0.5% BSA. The flow cytometric analysis was performed on a FACScalibur cytometer (BD Biosciences, San Jose, CA, USA) after acquisition of 10^5^ events. Exosomes-coated beads were primarily selected using forward and side scatter characteristics and fluorescence was analyzed using CellQuest software (BD Biosciences). Isotype-matched negative control antibodies were used in all the experiments. Exo-MSCs showed a positive expression for both markers (Figure S1B in Supplementary Material).

Exo-MSCs were slowly thawed prior to allogeneic intra-articular injections and used at the concentration of 500 μg protein/injection in a total volume of 500 μl. The exo-MSCs doses were chosen by extrapolating from our previous *in vitro* results (22).

### Hematological Analysis and Phenotypic Characterization of SF Lymphocytes

Synovial leukocytes were isolated from carpal joints just before intra-articular injections (at day 28) and 7 days after intra-articular injections (at day 35). A total of 0.5 ml of SF was aspirated and leukocytes were counted in an automatic hematology analyzer (Mindray BC-5300 Vet, Hamburg, Germany). The leukocytes were then isolated by centrifugation at 900 × *g* for 5 min and used for flow cytometry analysis or quantitative RT-PCR.

For flow cytometry, synovial leukocytes were stained with fluorescent-labeled mAbs against porcine CD3, CD4, CD8α, and CD16 (AbD Serotec, Kidlington, UK). The cytometric analysis was performed as follows: 2 × 10^5^ cells were incubated for 30 min at 4°C with appropriate concentrations of mAbs. The cells were washed and re-suspended in PBS. The flow cytometric analysis was performed in a FACScalibur cytometer (BD Biosciences) after acquisition of 10^5^ events. Cells were primarily selected using forward and side scatter characteristics and fluorescence was analyzed using CellQuest software (BD Biosciences, San Jose, CA, USA). Isotype-matched negative control antibodies were used in all the experiments.

### Quantitative RT-PCR

Total RNA was isolated from SF samples. The cDNA was synthesized from 1 μg of total RNA in a reverse transcription reaction for 1 h at 37°C using Superscript III reverse transcriptase (Thermo Fisher Scientific, Waltham, MA, USA). The sequences of the PCR primers were designed with the NCBI Primer-BLAST tool (www.ncbi.nlm.nih.gov/tools/primer-blast/). The primers used for gene expression studies in the porcine model are detailed in Table 1.

**Table 1:** **Sequences for the primers used in the quantitative qRT-PCR**.

Gene	Primers sequences
IL-1β	5′-GCACCTCTCAAGCAGAACAAAA-3′
	5′-CCTCTGGGTATGGCTTTCCTT-3′
IL-4	5′-GTCTGCTTACTGGCATGTACCA-3′
	5′-GCTCCATGCACGAGTTCTTTCT-3′
IL-6	5′-CCCCTAACCCCACCACAAAT-3′
	5′-AAGGCTGCGCAGGATGAG-3′
IL-8	5′-GCCAACACAACTTCAATCAAATCTA-3′
	5′-TGGGCATCCTGTGATTTCTCT-3′
IL-10	5′-CGGCGCTGTCATCAATTTCTG-3′
	5′-CCCCTCTCTTGGAGCTTGCTA-3′
TNF-α	5′-TCCCCTGTCCATCCCTTTATT-3′
	5′-CCAGCCCCTCATTCTCTTTCT-3′
TGF-β	5′-CCCAGAGTGGTTGTCCTTTGA-3′
	5′-GCGGAGCGTGTTATCTTTGCT-3′
β-2 microglobulin	5′-ACTTTTCACACCGCTCCAGT-3′
	5′-CGGATGGAACCCAGATACAT-3′

For transcriptional analysis, the RT-PCR products were quantified by the fluorescent method using the 2^−ΔCt^ expression. To normalize gene expression, two constitutively expressed genes were included (β-actin and β-2 microglobulin) and the most stable one, which was β-2 microglobulin, was used as housekeeping.

### Pressure Platform (PP) Gait Analysis

A 174.5 cm × 36.9 cm PP (Walkway™; Tekscan, South Boston, MA, USA), composed of individual sensors with a density of 1.4 sensor/cm^2^ and 9,152 sensors in total, was used for the biomechanical evaluation. The sensors of the PP walkway were calibrated according to the manufacturer’s specifications. Also, 7 days after intra-articular injections of BSA co-administered with PBS or exosomes, different kinetic parameters such as stance time, swing time, stride time, vertical maximum force, and impulse were monitored in the animal model. Kinetic gait analysis was performed prior to experimental procedures, and all the measurements were normalized and considering the gain weight of individual pigs.

### Statistical Analysis

Data were statistically analyzed using the Student’s *t*-test. The *p*-values ≤0.05 were considered statistically significant. All the statistical determinations were made using SPSS-21 software (SPSS, Chicago, IL, USA).

## Results

### Animal Model of Antigen-Induced Synovitis and Exosome-Based Therapy

**Figure 1:**
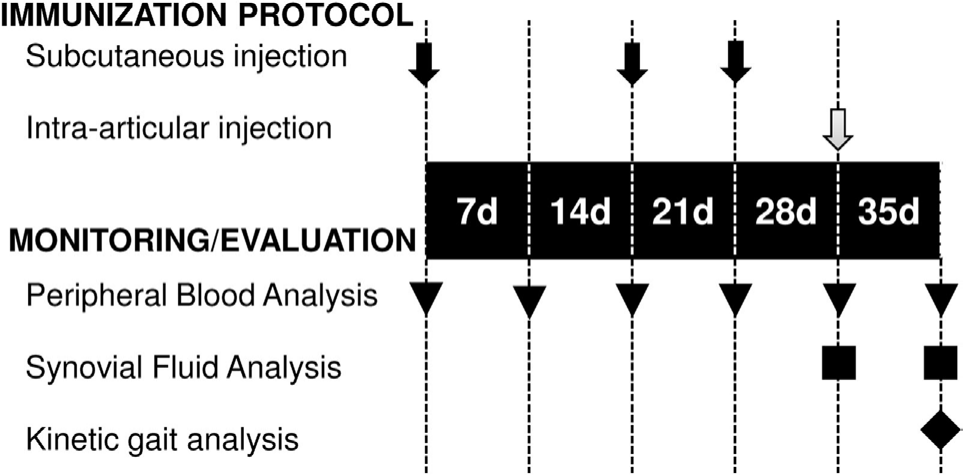
**Temporal scheme of the immunization protocol and monitoring**. Subcutaneous BSA injections (black arrows), intra-articular injections of BSA or BSA co-administered with exo-mesenchymal stem cells (gray arrow), blood sampling (triangles), synovial fluid sampling (squares), and kinetic gait analysis (rhombus) are shown.

An antigen-induced synovitis model was used to evaluate the therapeutic effect of exosome-based therapy. In our large animal model of synovitis, the BSA was intra-articularly injected to trigger an antigen-induced inflammation. The BSA was simultaneously co-administered with PBS or exo-MSCs. White blood cell (WBC) counts, differential cell counting, flow cytometry, gene expression of inflammatory cytokines, and kinetic parameters were evaluated at day 35. The immunization protocol and the monitoring of antigen-induced synovitis model are summarized in the Figure 1.

**Figure 2:**
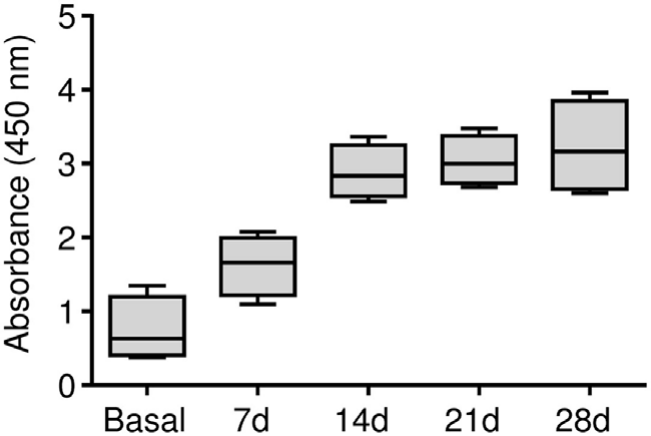
**Humoral response in the antigen-induced animal model of synovitis**. Plasma samples were weekly collected and anti-BSA IgG levels were quantified by ELISA immunoassay. The lower boundary of the box indicates the 25th percentile and the upper boundary the 75th percentile. Bars above and below the box indicate the 90th and 10th percentiles. The line within the box marks the median (*n* = 4).

Our results demonstrated that the BSA immunization protocol triggered a humoral response against BSA in this animal model, which is prerequisite to generate an antigen-induced synovitis. The anti-BSA IgG antibody titers were detected in all of the four animals and antibody concentrations significantly increased showing a maximum level at day 28 (Figure 2).

### SF Leukocytes and Differential Counts

On day 28, the BSA-immunized animals were anesthetized and a SF sample was aspirated to be used as basal reference for leukocyte counts. Once aspirated, the animals received an intra-articular injection of BSA to trigger a local inflammatory response. The BSA was co-administered with PBS (control joint) or with exo-MSCs. At day 7, after intra-articular injections, the SF from three animals was aspirated and analyzed by an automated hematological analyzer. As shown in Table 2, those animals which received an intra-articular injection of BSA showed a significant increase in terms of WBC counts when compared to basal samples. However, no differences were found between those joints where BSA was co-administered with PBS and those where BSA was co-administered with exo-MSCs. Interestingly, the differential cell counting revealed a statistically significant decrease of lymphocytes when BSA stimulation was counteracted by exo-MSCs.

**Table 2:** **White blood cell (WBC) counts, leukocyte distribution, and lymphocyte subsets on synovial fluids (SFs) from antigen-induced synovitis animal model**.

			Basal	BSA	BSA + exo-mesenchymal stem cells (MSCs)
SF	Leukocyte distribution	WBCs (×10^3^/μl)	0.753 ± 1.123	**2.348 ± 1.460^a^**	**3.030 ± 2.360^a^**
		Neutrophils (×10^3^/μl)	ND	0.608 ± 0.076	1.776 ± 1.783
		Lymphocytes (×10^3^/μl)	ND	2.371 ± 0.370	**0.992 ± 0.600^b^**
		Monocytes (×10^3^/μl)	ND	0.039 ± 0.067	0.209 ± 0.182
		Eosinophils (×10^3^/μl)	ND	0.202 ± 0.041	0.287 ± 0.423
		Basophils (×10^3^/μl)	ND	0.150 ± 0.064	0.049 ± 0.050
	Fluorescent activated cells sorting analysis	CD4+ CD8α− T cells (×10^3^/μl)	ND	0.171 ± 0.144	0.114 ± 0.112
		CD4− CD8α+ T cells (×10^3^/μl)	ND	0.476 ± 0.390	0.339 ± 0.291
		CD16+/CD8α− cells (×10^3^/μl)	ND	0.072 ± 0.045	0.379 ± 0.565
		CD16+/CD8α+ cells (×10^3^/μl)	ND	0.151 ± 0.102	0.140 ± 0.091

*SF samples were collected at day 28 just before intra-articular injections (basal). The BSA (co-administered with PBS or exo-MSCs) was injected at day 28 and SFs were collected at day 35. Synovial leukocytes were analyzed by automated hematological analyzer (*n* = 3). To identify the lymphocyte subsets, SF lymphocytes were isolated and analyzed by flow cytometry. Values represent the mean ± SD*.

*ND, non-detectable*.

*Bold values indicate significant differences*.

*^a^Statistically significant differences in a paired *t*-test when compared to basal level (*p* ≤ 0.05)*.

*^b^Statistically significant differences in a paired *t*-test when compared BSA and BSA + exo-MSCs (*p* ≤ 0.05)*.

Additionally, the SFs were centrifuged and synovial leukocytes were processed for flow cytometry analysis. The analysis of synovial lymphocytes was performed on CD4+/CD8α− T cells, CD4−/CD8α+ T cells, CD16+/CD8α− cells, and CD16+/CD8α+ cells. Our results did not show any significant difference when compared BSA co-administered with PBS and BSA co-administered with exo-MSCs (Table 2).

### Inflammatory Cytokines in SF

**Figure 3:**
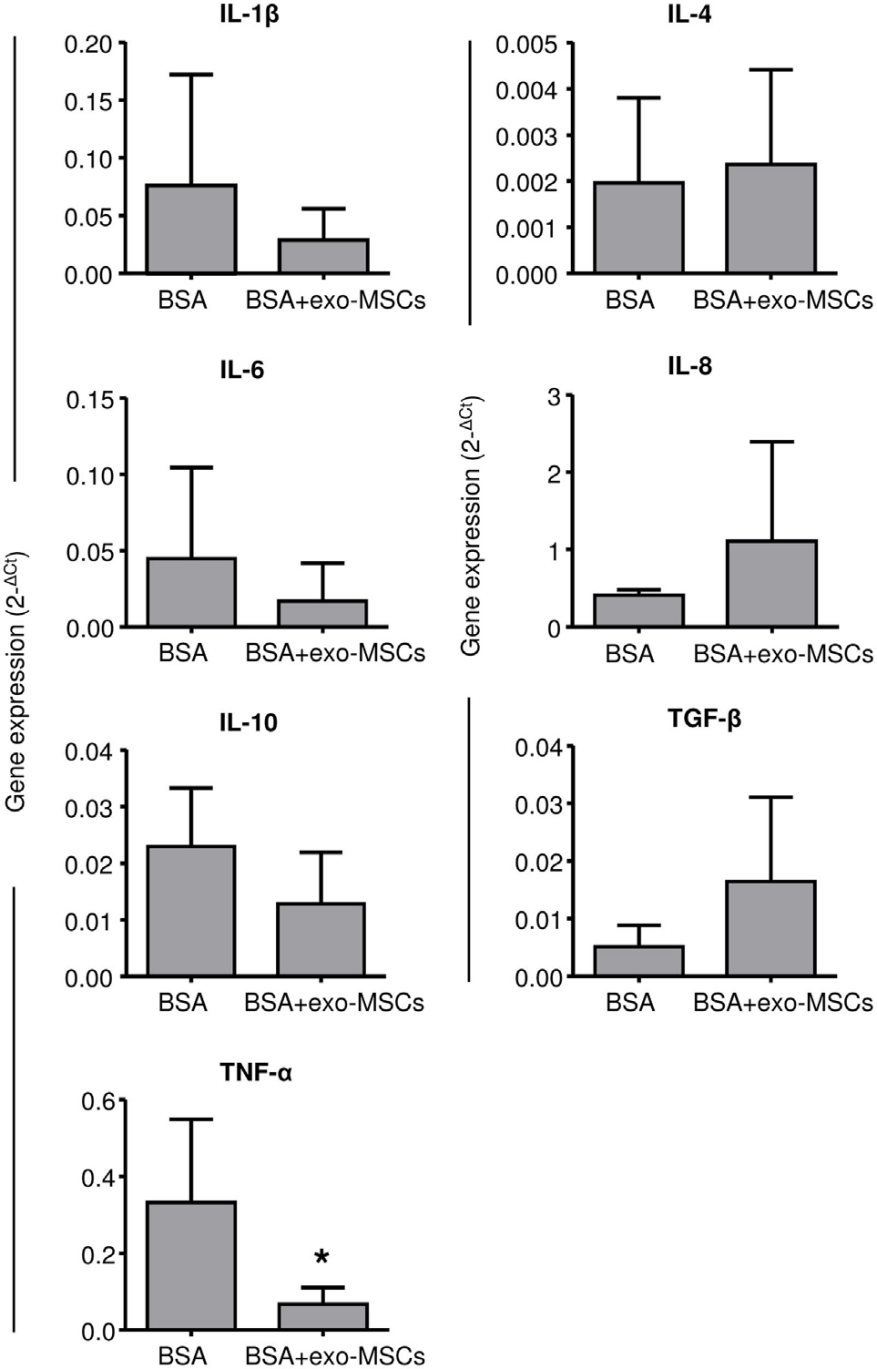
**Gene expression of cytokines in synovial fluid (SF)**. SF samples were collected 7 days after intra-articular injections and total RNA was isolated. The qRT-PCR products were quantified by the 2^ΔCt^ method using β-2 microglobulin as a housekeeping gene. Graph represents the mean ± SD of three independently performed experiments (*n* = 4). *Statistically significant difference in a paired *t*-test (*p* ≤ 0.05).

Once evaluated, the changes in the leukocyte counts as well as in the synovial lymphocytes, we aimed to evaluate the inflammatory environment by quantifying inflammatory cytokines by quantitative RT-PCR. Porcine-specific primers were designed to amplify IL-1β, IL-4, IL-6, IL-8, IL-10, TNFα, and TGF-β. As shown in Figure 3, the 2^−ΔCt^ values were compared between SFs where BSA was co-administered with PBS and SFs where BSA was co-administered with exo-MSCs. No differences were found in 7 out of 8 cytokines; however, a significant decrease (*p* = 0.05) was found for TNF-α when BSA was co-administered with exo-MSCs.

### Kinetic Gait Parameters on Animal Model

**Figure 4:**
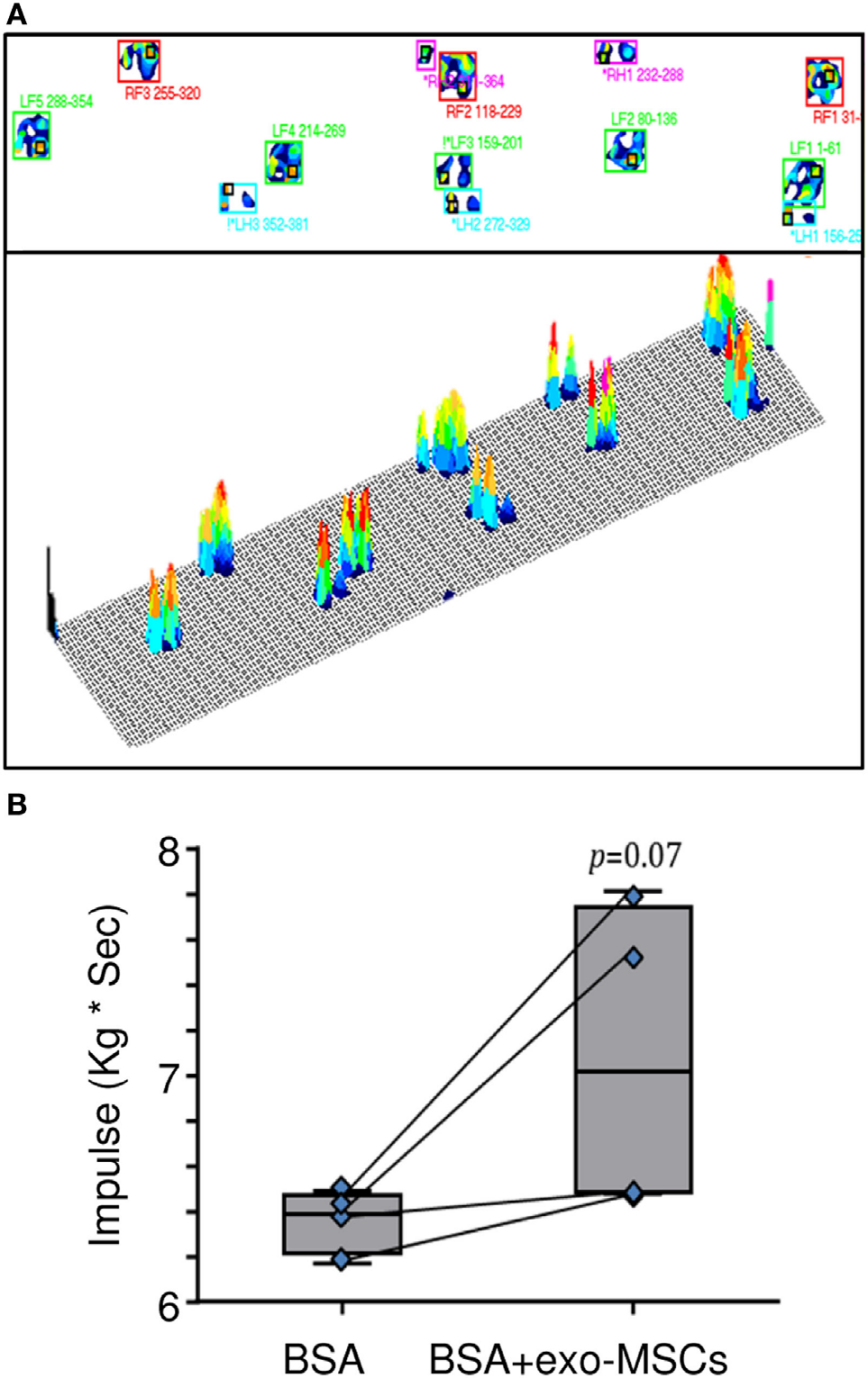
**Pressure platform (PP) gait analysis**. Then, 7 days after intra-articular injections of BSA or BSA co-administered with exo-mesenchymal stem cells (MSCs), a PP gait analysis was performed to evaluate plantar pressure distributions. **(A)** A representative image of the gait analysis (LF, left forelimb; LH, left hind limb; RF, right forelimb; RH, right hind limb) is represented. **(B)** Impulses in forelimbs with intra-articular BSA co-administered with PBS or exo-MSCs (*n* = 4). The lower boundary of the box indicates the 25th percentile and the upper boundary the 75th percentile. Bars above and below the box indicate the 90th and 10th percentiles. The line within the box marks the median. Measurements compared in a paired *t*-test.

The kinetic parameters were evaluated in a gait analysis system. This analysis allowed us the capture of kinetic, timing, and physical measurements. Different kinetic parameters such as stance time, swing time, stride time, vertical maximum force, and impulse were compared. As shown in Figure 4, a non-statistically significant trend to increase was found for the impulse in those joints where BSA was co-administered with exo-MSCs.

## Discussion

Synovitis is an inflammation of the synovial membrane, usually linked to osteoarthritis, rheumatoid arthritis or infections (41–43). It can be successfully treated with biologically based therapies such as platelet-rich plasma, or autologous conditioned serum (44, 45), or NSAIDs such as Ibuprofen or Naproxen (46). The MSCs-based therapies diminish the inflammatory response itself (12) and the intra-articular administration of MSCs has been found to reduce the nucleated cell counts in SF (47). Many authors have hypothesized that cell-free therapies may be safer and affordable than cell-based therapies (17, 18). In this sense, exo-MSCs have been considered as anti-inflammatory agents (48, 49) and a therapeutic alternative to cell-based therapies (50, 51).

The aim of this work was to evaluate the anti-inflammatory effect of exo-MSCs in a large animal model of synovitis. In this sense, our first sets of experiments were conducted to create an antigen-induced synovitis in a porcine model. This animal model has been immunologically characterized and our experience has demonstrated that it is particularly attractive in preclinical settings (34, 35), especially to evaluate the safety, feasibility, and dosage pattern of new therapies for synovitis.

The exo-MSCs used in this study were characterized by nanoparticle tracking analysis and flow cytometry. The mean size of these exosomes was 167.3 ± 2.6 nm. Although this size is larger to classically defined exosomes, it has been found that, in aqueous solution, exosomes are surrounded by a surface charge that may be the responsible for this larger size (52). This phenomenon has been previously described for nanoparticle tracking analyzed exosomes (53). Moreover, and due to the lack of porcine antibodies for CD9, CD63, and CD81 (classically used for exosomes characterization), CD44 and CD90 surface markers have been used to identify MSCs-derived extracellular vesicles (54).

In our antigen-induced synovitis model, a subcutaneous pre-sensitization with BSA was required before the intra-articular injection of BSA. This pre-sensitization induced a potent humoral response, which was found to be as effective as previously described antigen-induced synovitis in rabbits and dogs (55, 56). These BSA-presensitized animals received an intra-articular injection of BSA, which triggered a local inflammatory response with a significant increase of WBCs in SF. It is important to note that the WBC count is one of the most frequent tests in the analysis of SF. In this sense, human SFs with less than 200 cells/μl are classified as “normal” and those with less than 2,000 cells/μl are classified as “non-inflammatory.” In our antigen-induced synovitis model, those animals that received an intra-articular injection of BSA showed a WBC infiltration that can be classified as “inflammatory SF” (41).

Once demonstrated that intra-articular BSA triggered a local inflammatory reaction, we aimed to counteract this reaction by an intra-articular administration of exo-MSCs. As shown in the results section, no differences were found in terms of WBCs when exo-MSCs were co-administered with BSA. However, the differential cell count of leukocytes showed statistically significant differences in the lymphocyte counts being lower in those joints where BSA was co-administered with exo-MSCs. Based on that, here, we assume that exo-MSCs efficiently counteracted the antigen-driven T cell response and point out that these exosomes may represent a therapeutic strategy for the treatment of T cell-mediated diseases such as rheumatoid arthritis. These *in vivo* results are in agreement with *in vitro* results using stimulated T cells co-cultured with human exosomes from adipose-derived stem cells (22). In these studies, the effect of exo-MSCs on proliferative, differentiation, and functional behavior of T cells was significantly modified by exosomes.

Additionally, here, we hypothesized that exosomes may also inhibit or decrease the production of pro-inflammatory cytokines. In order to evaluate the inflammatory reaction after intra-articular BSA injections, a qRT-PCR analysis was performed for several cytokines (IL-1β, IL-4, TGF-β, IL-8, IL-10, TNF-α, and IL-6). This analysis revealed some differences in IL-1β, IL-8, and TNF-α after intra-articular injections of BSA (co-administered with PBS or with exo-MSCs).

The IL-1β is a critical mediator of osteoarthritis and the intra-articular injection of this recombinant cytokine has been used to induce a transient inflammatory response in an experimentally induced synovitis (57). In the case of IL-8, this chemokine participates to the inflammatory process in the early synovitis of rheumatoid arthritis (58), and similar to IL-1β, the intra-articular administration has been also used to induce acute synovitis in rabbits (59). Finally, the level of TNF-α in synovia has been correlated with pain and disease progression (60, 61) and anti-TNF-α agents have been widely used for the treatment of active rheumatoid arthritis (62, 63).

Based on the above-described relations between IL-1β, IL-8, TNF-α, and synovitis progression, we aimed to quantify their gene expression in SFs when exo-MSCs were co-administered in the joints. Uniquely, the TNF-α level was found to be statistically decreased by the co-administration of exo-MSCs. We suggest that this result is very relevant considering that TNF-α is a therapeutic target for the treatment of inflammatory diseases (64). Indeed, targeted treatments against synovitis and rheumatoid arthritis have been efficiently developed against this cytokine (65).

Regarding to the not statistically significant increase of IL-8 observed with exo-MSCs, it is important to note that this chemokine is abundantly secreted by MSCs from adipose tissue and bone marrow (66). More importantly, this chemokine has been found to be contained in exosomes derived from umbilical cord-MSCs (67) as well as in menstrual blood-MSCs (68). Based on these observations, we assume that the increase of IL-8 is the consequence of an exogenous administration of IL-8 linked to exo-MSCs. Additionally, taking into account that IL-8 is one of the most potent chemoattractant molecule for neutrophils (69), this may also explain the increase (although not significant) of infiltrated neutrophils in SFs treated with exo-MSCs.

It is important to note that the determination of the different cytokines in SF was firstly addressed with a multiplexed immunoassay by Luminex xMAP technology using the ProcartaPlex Porcine Cytokine and Chemokine Panel 1 (eBioscience, San Diego, CA, USA). The following cytokines were measured: IFNα, IFNγ, IL-1β, IL-4, IL-6, IL-8, IL-10, IL-12p40, and TNFα. The cytokine analysis demonstrated that, probably because of the detection limit of commercially available swine immune reagents or because the evaluation time point was too short for protein translation, eight out of nine cytokines were undetectable in SFs with this technique. Uniquely, the IL-12p40 could be detected in all the samples but no differences were found between groups.

Although this paper has not been focused on the biological mechanisms, which promote the decrease of TNF-α in the synovitis model, the bibliography has several references that support the association between exo-MSCs and TNF-α. As an example, recent studies have demonstrated that exosomes derived from human umbilical cord MSCs reduced the TNF-α release from CD3/CD28-stimulated PBLs (70). Moreover, exosomes from bone marrow-derived MSCs also suppressed the secretion of TNF-α from T cells (71). More recently, the immunomodulatory effect of these exosomes against TNF-α transcription was demonstrated *in vivo* in an experimental colitis model. In this animal model, the intravenous injection of exosomes from bone marrow-derived MSCs reduced the TNF-α in injured colon (72). Altogether, these *in vitro* and *in vivo* studies are in agreement with our results and support the immunomodulatory effect of these exosomes in the animal model.

Finally, this paper aimed to evaluate the therapeutic effect of exo-MSCs in a functional kinetic assessment. Apart from the analysis of synovial leukocyte subsets and inflammatory cytokines in the animal model, different kinetic parameters such as stance time, swing time, stride time, vertical maximum force, and impulse were monitored in the animal model by PP gait analysis (73). The quadruped gait analysis demonstrated that exo-MSCs co-administered with the BSA had a non-significant trend for the improvement of the impulse. This trend may indicate a pain reduction linked to the anti-inflammatory effect of exo-MSCs. Finally, it is important to note that the absence of statistical differences would not necessarily imply that the kinetic was unaffected; since the pressure gait was performed under analgesia due to ethical consideration and animal welfare guidelines. In the same way, due to ethical limitations, animals could only be evaluated at day 7 after treatment. In this sense, this study can be considered a preliminary approach and further studies will be performed to evaluate the changes in a long-term study, including the histological follow-up of the lesions as well as the cytokine quantification using immunoassays.

In conclusion, to our knowledge, this is the first report describing the use of exo-MSCs for the treatment of synovitis in a large animal model. The decrease of synovial lymphocytes, the downregulation of TNF-α transcripts, as well as the trend to improve the impulse in exosome-treated joints, point out that exosomes may represent a promising therapeutic option for the treatment of synovitis.

## Author Contributions

RB, FS-M, and FJV equally contributed and should be regarded as co-first authors. FS-M, RT, and JGC conceived and designed the experiments. FJV, VA, and RB performed the experiments and analyzed the data. VA, FS-M, RT, and JGC wrote the manuscript.

## Conflict of Interest Statement

The authors declare that the research was conducted in the absence of any commercial or financial relationships that could be construed as a potential conflict of interest. The reviewer AL-C and handling Editor declared their shared affiliation, and the handling Editor states that the process nevertheless met the standards of a fair and objective review.

## Supplementary Material

Figure S1 **Characterization of exo-MSCs**. Panel **(A)** shows the frequency size distribution graph of exo-MSCs. The nanoparticle tracking analysis was performed on exosomes samples to quantify size distribution and particle concentration (*n* = 3). Error bars indicate ±1 SEM. Panel **(B)** shows the expression of CD44 and CD90 in exosomes-coated latex beads. Exosomes adhered to latex beads were analyzed by multicolor flow cytometry. Representative histograms of CD44 and CD90 expression are shown (filled histograms) together with their negative control (gray lined histograms).

## References

[1] Scanzello CR Goldring SR. The role of synovitis in osteoarthritis pathogenesis. Bone (2012) 51:249–57. 10.1016/j.bone.2012.02.012 22387238 PMC3372675

[2] Arend WP Firestein GS. Pre-rheumatoid arthritis: predisposition and transition to clinical synovitis. Nat Rev Rheumatol (2012) 8:573–86. 10.1038/nrrheum.2012.134 22907289

[3] McIlwraith CW Use of synovial fluid and serum biomarkers in equine bone and joint disease: a review. Equine Vet J (2005) 37:473–82. 10.2746/042516405774480102 16163952

[4] McIlwraith CW Frisbie DD Rodkey WG Kisiday JD Werpy NM Kawcak CE Evaluation of intra-articular mesenchymal stem cells to augment healing of microfractured chondral defects. Arthroscopy (2011) 27:1552–61. 10.1016/j.arthro.2011.06.002 21862278

[5] Kofoed H. Synovitis causes hypoxia and acidity in synovial fluid and subchondral bone. Injury (1986) 17:391–4. 10.1016/0020-1383(86)90078-1 3102376

[6] Walsh DA. Angiogenesis in osteoarthritis and spondylosis: successful repair with undesirable outcomes. Curr Opin Rheumatol (2004) 16:609–15. 10.1097/01.bor.0000133662.60223.ee 15314503

[7] Zayat AS Conaghan PG Sharif M Freeston JE Wenham C Hensor EMA Do non-steroidal anti-inflammatory drugs have a significant effect on detection and grading of ultrasound-detected synovitis in patients with rheumatoid arthritis? Results from a randomised study. Ann Rheum Dis (2011) 70:1746–51. 10.1136/annrheumdis-2011-200017 21803749

[8] Liberman B Herman A Schindler A Sherr-Lurie N Ganel A Givon U. The value of hip aspiration in pediatric transient synovitis. J Pediatr Orthop (2013) 33:124–7. 10.1097/BPO.0b013e31827268b8 23389564

[9] Van den Bekerom MPJ Struijs PAA Blankevoort L Welling L van Dijk CN Kerkhoffs GM. What is the evidence for rest, ice, compression, and elevation therapy in the treatment of ankle sprains in adults? J Athl Train (2012) 47:435–43. 10.4085/1062-6050-47.4.14 22889660 PMC3396304

[10] Xie X Zhang C Tuan RS. Biology of platelet-rich plasma and its clinical application in cartilage repair. Arthritis Res Ther (2014) 16:204. 10.1186/ar4493 25164150 PMC3978832

[11] Rutgers M Creemers LB Auw Yang KG Raijmakers NJH Dhert WJA Saris DBF. Osteoarthritis treatment using autologous conditioned serum after placebo. Acta Orthop (2015) 86:114–8. 10.3109/17453674.2014.950467 25140983 PMC4366668

[12] De Witte SFH Franquesa M Baan CC Hoogduijn MJ Toward development of iMesenchymal stem cells for immunomodulatory therapy. Front Immunol (2015) 6:648 10.3389/fimmu.2015.00648 26779185 PMC4701910

[13] Jorgensen C Noël D. Mesenchymal stem cells in osteoarticular diseases. Regen Med (2011) 6:44–51. 10.2217/rme.11.80 21999261

[14] Pers Y-M Jorgensen C. Adipose derived stem cells for regenerative therapy in osteoarticular diseases. Horm Mol Biol Clin Investig (2016) 28:113–20. 10.1515/hmbci-2016-0010 27092656

[15] Pigott JH Ishihara A Wellman ML Russell DS Bertone AL. Investigation of the immune response to autologous, allogeneic, and xenogeneic mesenchymal stem cells after intra-articular injection in horses. Vet Immunol Immunopathol (2013) 156:99–106. 10.1016/j.vetimm.2013.09.003 24094688

[16] Williams AR Suncion VY McCall F Guerra D Mather J Zambrano JP Durable scar size reduction due to allogeneic mesenchymal stem cell therapy regulates whole-chamber remodeling. J Am Heart Assoc (2013) 2:e000140. 10.1161/JAHA.113.000140 23686370 PMC3698774

[17] De Jong OG Van Balkom BWM Schiffelers RM Bouten CVC Verhaar MC. Extracellular vesicles: potential roles in regenerative medicine. Front Immunol (2014) 5:608. 10.3389/fimmu.2014.00608 25520717 PMC4253973

[18] Zhang B Yin Y Lai RC Lim SK Immunotherapeutic potential of extracellular vesicles. Front Immunol (2014) 5:518 10.3389/fimmu.2014.00518 25374570 PMC4205852

[19] Merino AM Hoogduijn MJ Borras FE Franquesa M Therapeutic potential of extracellular vesicles. Front Immunol (2014) 5:658 10.3389/fimmu.2014.00658 25566267 PMC4271725

[20] Ludwig A Kordelas L Rebmann V Radtke S Felderhoff-Müser U Horn P Exosomes – from bench to bedside. Klin Pädiatr (2012) 224:A6 10.1055/s-0032-1330775

[21] Mathivanan S Simpson RJ. ExoCarta: a compendium of exosomal proteins and RNA. Proteomics (2009) 9:4997–5000. 10.1002/pmic.200900351 19810033

[22] Blazquez R Sanchez-Margallo FM de la Rosa O Dalemans W Alvarez V Tarazona R Immunomodulatory potential of human adipose mesenchymal stem cells derived exosomes on in vitro stimulated T cells. Front Immunol (2014) 5:556. 10.3389/fimmu.2014.00556 25414703 PMC4220146

[23] Teng X Chen L Chen W Yang J Yang Z Shen Z. Mesenchymal stem cell-derived exosomes improve the microenvironment of infarcted myocardium contributing to angiogenesis and anti-inflammation. Cell Physiol Biochem (2015) 37:2415–24. 10.1159/000438594 26646808

[24] Yu B Kim HW Gong M Wang J Millard RW Wang Y Exosomes secreted from GATA-4 overexpressing mesenchymal stem cells serve as a reservoir of anti-apoptotic microRNAs for cardioprotection. Int J Cardiol (2015) 182:349–60. 10.1016/j.ijcard.2014.12.043 25590961 PMC4382384

[25] Zhou Y Xu H Xu W Wang B Wu H Tao Y Exosomes released by human umbilical cord mesenchymal stem cells protect against cisplatin-induced renal oxidative stress and apoptosis in vivo and in vitro. Stem Cell Res Ther (2013) 4:34. 10.1186/scrt194 23618405 PMC3707035

[26] Shabbir A Cox A Rodriguez-Menocal L Salgado M Van Badiavas E. Mesenchymal stem cell exosomes induce proliferation and migration of normal and chronic wound fibroblasts, and enhance angiogenesis in vitro. Stem Cells Dev (2015) 24:1635–47. 10.1089/scd.2014.0316 25867197 PMC4499790

[27] Lai RC Arslan F Lee MM Sze NSK Choo A Chen TS Exosome secreted by MSC reduces myocardial ischemia/reperfusion injury. Stem Cell Res (2010) 4:214–22. 10.1016/j.scr.2009.12.003 20138817

[28] Wang B Yao K Huuskes BM Shen H-H Zhuang J Godson C Mesenchymal stem cells deliver exogenous microRNA-let7c via exosomes to attenuate renal fibrosis. Mol Ther (2016) 24:1290–301. 10.1038/mt.2016.90 27203438 PMC5088767

[29] Yuan H Guan J Zhang J Zhang R Li M. Exosomes secreted by human urine-derived stem cells accelerate skin wound healing by promoting angiogenesis in rat. Cell Biol Int (2016). 10.1002/cbin.10615 27098397

[30] Rager TM Olson JK Zhou Y Wang Y Besner GE. Exosomes secreted from bone marrow-derived mesenchymal stem cells protect the intestines from experimental necrotizing enterocolitis. J Pediatr Surg (2016) 51:942–7. 10.1016/j.jpedsurg.2016.02.061 27015901 PMC4921266

[31] Monsel A Zhu Y-G Gudapati V Lim H Lee JW. Mesenchymal stem cell derived secretome and extracellular vesicles for acute lung injury and other inflammatory lung diseases. Expert Opin Biol Ther (2016) 16:859–71. 10.1517/14712598.2016.1170804 27011289 PMC5280876

[32] Doeppner TR Herz J Görgens A Schlechter J Ludwig A-K Radtke S Extracellular vesicles improve post-stroke neuroregeneration and prevent postischemic immunosuppression. Stem Cells Transl Med (2015) 4:1131–43. 10.5966/sctm.2015-0078 26339036 PMC4572905

[33] Ophelders DR Wolfs TG Jellema RK Zwanenburg A Andriessen P Delhaas T Mesenchymal stromal cell-derived extracellular vesicles protect the fetal brain after hypoxia-ischemia. Stem Cells Transl Med (2016) 5:754–63. 10.5966/sctm.2015-0197 27160705 PMC4878333

[34] Álvarez V Sánchez-Margallo F-M Blázquez R Tarazona R Casado JG Comparison of mesenchymal stem cells and leukocytes from large white and Göttingen Minipigs: clues for stem cell-based immunomodulatory therapies. Vet Immunol Immunopathol (2016) 179:63–9. 10.1016/j.vetimm.2016.08.002 27590427

[35] Casado JG Gomez-Mauricio G Alvarez V Mijares J Tarazona R Bernad A Comparative phenotypic and molecular characterization of porcine mesenchymal stem cells from different sources for translational studies in a large animal model. Vet Immunol Immunopathol (2012) 147:104–12. 10.1016/j.vetimm.2012.03.015 22521281

[36] El-Andaloussi S Lee Y Lakhal-Littleton S Li J Seow Y Gardiner C Exosome-mediated delivery of siRNA in vitro and in vivo. Nat Protoc (2012) 7:2112–26. 10.1038/nprot.2012.131 23154783

[37] Roccaro AM Sacco A Maiso P Azab AK Tai Y-T Reagan M BM mesenchymal stromal cell-derived exosomes facilitate multiple myeloma progression. J Clin Invest (2013) 123:1542–55. 10.1172/JCI66517 23454749 PMC3613927

[38] Kruger S Abd Elmageed ZY Hawke DH Wörner PM Jansen DA Abdel-Mageed AB Molecular characterization of exosome-like vesicles from breast cancer cells. BMC Cancer (2014) 14:44. 10.1186/1471-2407-14-44 24468161 PMC3936808

[39] Wang J Yao Y Wu J Li G. Identification and analysis of exosomes secreted from macrophages extracted by different methods. Int J Clin Exp Pathol (2015) 8:6135–42. 26261491 PMC4525825

[40] Théry C Amigorena S Raposo G Clayton A. Isolation and characterization of exosomes from cell culture supernatants and biological fluids. Curr Protoc Cell Biol (2006) 22:1–29. 10.1002/0471143030.cb0322s30 18228490

[41] El-Gabalawy H The challenge of early synovitis: multiple pathways to a common clinical syndrome. Arthritis Res (1999) 1:31–6. 10.1186/ar8 11094411 PMC128867

[42] Kastrissianakis K Beattie TF. Transient synovitis of the hip: more evidence for a viral aetiology. Eur J Emerg Med (2010) 17:270–3. 10.1097/MEJ.0b013e32832b1664 20523221

[43] Terao C Hashimoto M Yamamoto K Murakami K Ohmura K Nakashima R Three groups in the 28 joints for rheumatoid arthritis synovitis – analysis using more than 17,000 assessments in the KURAMA database. PLoS One (2013) 8:e59341 10.1371/journal.pone.0059341 23555018 PMC3595245

[44] Baltzer AWA Moser C Jansen SA Krauspe R. Autologous conditioned serum (Orthokine) is an effective treatment for knee osteoarthritis. Osteoarthritis Cartilage (2009) 17:152–60. 10.1016/j.joca.2008.06.014 18674932

[45] Lippross S Moeller B Haas H Tohidnezhad M Steubesand N Wruck CJ Intraarticular injection of platelet-rich plasma reduces inflammation in a pig model of rheumatoid arthritis of the knee joint. Arthritis Rheum (2011) 63:3344–53. 10.1002/art.30547 21769848

[46] Nouri A Walmsley D Pruszczynski B Synder M Transient synovitis of the hip: a comprehensive review. J Pediatr Orthop B (2014) 23:32–6. 10.1097/BPB.0b013e328363b5a3 23812087

[47] Williams LB Koenig JB Black B Gibson TWG Sharif S Koch TG Equine allogeneic umbilical cord blood derived mesenchymal stromal cells reduce synovial fluid nucleated cell count and induce mild self-limiting inflammation when evaluated in an LPS induced synovitis model. Equine Vet J (2015) 48:619–25. 10.1111/evj.12477 26114736

[48] Yu B Zhang X Li X Exosomes derived from mesenchymal stem cells. Int J Mol Sci (2014) 15:4142–57. 10.3390/ijms15034142 24608926 PMC3975389

[49] Zhang B Yin Y Lai RC Tan SS Choo ABH Lim SK. Mesenchymal stem cells secrete immunologically active exosomes. Stem Cells Dev (2014) 23:1233–44. 10.1089/scd.2013.0479 24367916

[50] Barile L Moccetti T Marbán E Vassalli G Roles of exosomes in cardioprotection. Eur Heart J (2016) ehw304 10.1093/eurheartj/ehw304 27443883

[51] Ibrahim AG-E Cheng K Marbán E. Exosomes as critical agents of cardiac regeneration triggered by cell therapy. Stem Cell Reports (2014) 2:606–19. 10.1016/j.stemcr.2014.04.006 24936449 PMC4050492

[52] Chernyshev VS Rachamadugu R Tseng YH Belnap DM Jia Y Branch KJ Size and shape characterization of hydrated and desiccated exosomes. Anal Bioanal Chem (2015) 407:3285–301. 10.1007/s00216-015-8535-3 25821114

[53] Gallet R Dawkins J Valle J Simsolo E de Couto G Middleton R Exosomes secreted by cardiosphere-derived cells reduce scarring, attenuate adverse remodelling, and improve function in acute and chronic porcine myocardial infarction. Eur Heart J (2017) 38(3):201–11. 10.1093/eurheartj/ehw240 28158410 PMC5837390

[54] L Ramos T Sánchez-Abarca LI Muntión S Preciado S Puig N López-Ruano G MSC surface markers (CD44, CD73, and CD90) can identify human MSC-derived extracellular vesicles by conventional flow cytometry. Cell Commun Signal (2016) 14:2. 10.1186/s12964-015-0124-8 26754424 PMC4709865

[55] Goldlust MB Rich LC Brown WR. Immune synovitis in rabbits. Effects of differing schedules for intra-articular challenge with antigen. Am J Pathol (1978) 91:329–44. 645828 PMC2018194

[56] Ohashi F Shimada T Sakurai M Ishihara S Kuwamura M Yamate J The production of arthritis in beagles by an immunological reaction to bovine serum albumin. Exp Anim (1996) 45:299–307. 10.1538/expanim.45.299 8902492

[57] Ross TN Kisiday JD Hess T McIlwraith CW. Evaluation of the inflammatory response in experimentally induced synovitis in the horse: a comparison of recombinant equine interleukin 1 beta and lipopolysaccharide. Osteoarthritis Cartilage (2012) 20:1583–90. 10.1016/j.joca.2012.08.008 22917743

[58] Takahashi Y Kasahara T Sawai T Rikimaru A Mukaida N Matsushima K The participation of IL-8 in the synovial lesions at an early stage of rheumatoid arthritis. Tohoku J Exp Med (1999) 188:75–87. 10.1620/tjem.188.75 10494903

[59] Endo H Akahoshi T Nishimura A Tonegawa M Takagishi K Kashiwazaki S Experimental arthritis induced by continuous infusion of IL-8 into rabbit knee joints. Clin Exp Immunol (1994) 96:31–5. 10.1111/j.1365-2249.1994.tb06225.x 7512008 PMC1534528

[60] Güven O Tekin U Salmanoğlu B Kaymak E. Tumor necrosis factor-alpha levels in the synovial fluid of patients with temporomandibular joint internal derangement. J Craniomaxillofac Surg (2015) 43:102–5. 10.1016/j.jcms.2014.10.017 25465487

[61] Santos Savio A Machado Diaz AC Chico Capote A Miranda Navarro J Rodríguez Alvarez Y Bringas Pérez R Differential expression of pro-inflammatory cytokines IL-15Ralpha, IL-15, IL-6 and TNFalpha in synovial fluid from rheumatoid arthritis patients. BMC Musculoskelet Disord (2015) 16:51. 10.1186/s12891-015-0516-3 25879761 PMC4359511

[62] Bazzani C Filippini M Caporali R Bobbio-Pallavicini F Favalli EG Marchesoni A Anti-TNFalpha therapy in a cohort of rheumatoid arthritis patients: clinical outcomes. Autoimmun Rev (2009) 8:260–5. 10.1016/j.autrev.2008.11.001 19027090

[63] Reid A Brady A Blake C Mongey A-B Veale DJ FitzGerald O Randomised controlled trial examining the effect of exercise in people with rheumatoid arthritis taking anti-TNFα therapy medication. BMC Musculoskelet Disord (2011) 12:11. 10.1186/1471-2474-12-11 21232112 PMC3024271

[64] Esposito E Cuzzocrea S. TNF-alpha as a therapeutic target in inflammatory diseases, ischemia-reperfusion injury and trauma. Curr Med Chem (2009) 16:3152–67. 10.2174/092986709788803024 19689289

[65] Boissier M-C Semerano L Challal S Saidenberg-Kermanac’h N Falgarone G. Rheumatoid arthritis: from autoimmunity to synovitis and joint destruction. J Autoimmun (2012) 39:222–8. 10.1016/j.jaut.2012.05.021 22704962

[66] Kyurkchiev D Bochev I Ivanova-Todorova E Mourdjeva M Oreshkova T Belemezova K Secretion of immunoregulatory cytokines by mesenchymal stem cells. World J Stem Cells (2014) 6:552–70. 10.4252/wjsc.v6.i5.552 25426252 PMC4178255

[67] Zhang B Shen L Shi H Pan Z Wu L Yan Y Exosomes from human umbilical cord mesenchymal stem cells: identification, purification, and biological characteristics. Stem Cells Int (2016) 2016:1929536. 10.1155/2016/1929536 28105054 PMC5220513

[68] Chen L Xiang B Wang X Xiang C. Exosomes derived from human menstrual blood-derived stem cells alleviate fulminant hepatic failure. Stem Cell Res Ther (2017) 8:9. 10.1186/s13287-016-0453-6 28115012 PMC5260032

[69] De Oliveira S Reyes-Aldasoro CC Candel S Renshaw SA Mulero V Calado A. Cxcl8 (IL-8) mediates neutrophil recruitment and behavior in the zebrafish inflammatory response. J Immunol (2013) 190:4349–59. 10.4049/jimmunol.1203266 23509368 PMC3736093

[70] Liu M Wang J Liu M Hu X Xu J. [Study of immunomodulatory function of exosomes derived from human umbilical cord mesenchymal stem cells]. Zhonghua Yi Xue Za Zhi (2015) 95:2630–3. 10.3760/cma.j.issn.0376-2491.2015.32.014 26711615

[71] Chen W Huang Y Han J Yu L Li Y Lu Z Immunomodulatory effects of mesenchymal stromal cells-derived exosome. Immunol Res (2016) 64:831–40. 10.1007/s12026-016-8798-6 27115513

[72] Yang J Liu X-X Fan H Tang Q Shou Z-X Zuo D-M Extracellular vesicles derived from bone marrow mesenchymal stem cells protect against experimental colitis via attenuating colon inflammation, oxidative stress and apoptosis. PLoS One (2015) 10:e0140551. 10.1371/journal.pone.0140551 26469068 PMC4607447

[73] Lascelles BDX Roe SC Smith E Reynolds L Markham J Marcellin-Little D Evaluation of a pressure walkway system for measurement of vertical limb forces in clinically normal dogs. Am J Vet Res (2006) 67:277–82. 10.2460/ajvr.67.2.277 16454633

