# Supplementary figures and images for: Mesenchymal Stem Cell-Derived Exosomes: Immunomodulatory Evaluation in an Antigen-Induced Synovitis Porcine Model
Source: Front Vet Sci. 2017 Mar 21;4:39. doi: 10.3389/fvets.2017.00039 (PMC5359696; doi:10.3389/fvets.2017.00039)

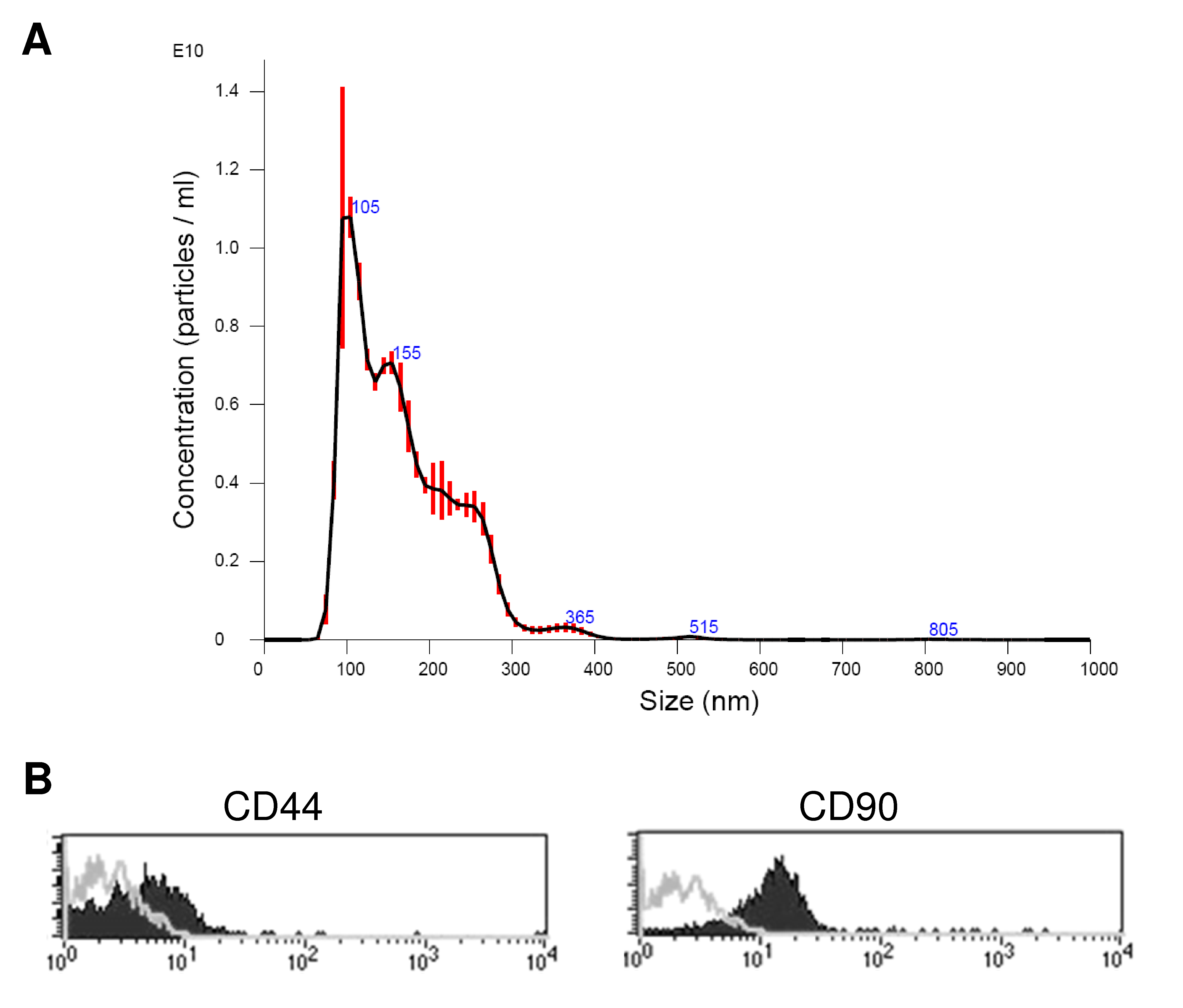

Supplement: Figure S1 — Characterization of exo-MSCs. Panel (A) shows the frequency size distribution graph of exo-MSCs. The nanoparticle tracking analysis was performed on exosomes samples to quantify size distribution and particle concentration (n = 3). Error bars indicate ±1 SEM. Panel (B) shows the expression of CD44 and CD90 in exosomes-coated latex beads. Exosomes adhered to latex beads were analyzed by multicolor flow cytometry. Representative histograms of CD44 and CD90 expression are shown (filled histograms) together with their negative control (gray lined histograms). [file Image_1.tif]
